# Computation Offloading and Resource Allocation Based on P-DQN in LEO Satellite Edge Networks

**DOI:** 10.3390/s23249885

**Published:** 2023-12-17

**Authors:** Xu Yang, Hai Fang, Yuan Gao, Xingjie Wang, Kan Wang, Zheng Liu

**Affiliations:** 1 Xi’an Institute of Space Radio Technology, Xi’an 710100, China; yangx@cast504.com (X.Y.); fangh@cast504.com (H.F.); gaoy69@cast504.com (Y.G.); 2 School of Computer Science and Engineering, Xi’an University of Technology, Xi’an 710048, China; wangkan@xaut.edu.cn (K.W.); liuzheng@xaut.edu.cn (Z.L.)

**Keywords:** LEO satellite edge networks, offloading decision, resource allocation, hybrid action space, P-DQN

## Abstract

Traditional low earth orbit (LEO) satellite networks are typically independent of terrestrial networks, which develop relatively slowly due to the on-board capacity limitation. By integrating emerging mobile edge computing (MEC) with LEO satellite networks to form the business-oriented “end-edge-cloud” multi-level computing architecture, some computing-sensitive tasks can be offloaded by ground terminals to satellites, thereby satisfying more tasks in the network. How to make computation offloading and resource allocation decisions in LEO satellite edge networks, nevertheless, indeed poses challenges in tracking network dynamics and handling sophisticated actions. For the discrete-continuous hybrid action space and time-varying networks, this work aims to use the parameterized deep Q-network (P-DQN) for the joint computation offloading and resource allocation. First, the characteristics of time-varying channels are modeled, and then both communication and computation models under three different offloading decisions are constructed. Second, the constraints on task offloading decisions, on remaining available computing resources, and on the power control of LEO satellites as well as the cloud server are formulated, followed by the maximization problem of satisfied task number over the long run. Third, using the parameterized action Markov decision process (PAMDP) and P-DQN, the joint computing offloading, resource allocation, and power control are made in real time, to accommodate dynamics in LEO satellite edge networks and dispose of the discrete-continuous hybrid action space. Simulation results show that the proposed P-DQN method could approach the optimal control, and outperforms other reinforcement learning (RL) methods for merely either discrete or continuous action space, in terms of the long-term rate of satisfied tasks.

## 1. Introduction

With the growth in global communications demand and the development of space Internet, connectivity to rural areas has become imperative for future networks. Since the traditional terrestrial network has limited coverage in remote areas, its infrastructure is vulnerable to natural disasters, e.g., earthquakes and floods, thus disrupting user communications [1]. Therefore, it is a prerequisite to support lower latency and more reliable communication in future wireless networks [2].

In the past few decades, satellite and terrestrial networks typically developed independently and competed with each other [3]. Although the terrestrial network is advantageous in terms of high-speed data transmission and low latency, its coverage is limited, covering only about 6% of the Earth’s surface and about 20% of the land area [4]. In contrast, satellite networks are not subject to regional restrictions and can cover the globe, meeting the Internet needs in remote areas, sea and air. Besides, satellite networks have higher survival when disasters occur, especially in earthquakes, yet also face the challenge of long-distance transmission.

Therefore, both industry and academia are promoting the integration of terrestrial and satellite communications, to achieve seamless coverage and high-quality service anytime and anywhere. It is apparent that global seamless communication will be an important component in 6G networks, and thus both academia and industry have begun to discuss its requirements, application scenarios, and potential solutions [5,6,7,8].

The 6G network will form a three-dimensional coverage of global communication through the interaction of satellite and terrestrial networks, forming a seamless three-dimensional coverage on a global scale, and is expected to provide heterogeneous services and seamless network coverage [9,10]. The integrated satellite-terrestrial network architecture can integrate the information of both networks, thereby ensuring wider network coverage and higher performance [11,12]. Yet, when providing ubiquitous and reliable services, the integrated satellite–terrestrial network also faces challenges, especially in meeting the growing quality of service (QoS) requirement. That is, with the rapid development of computing-intensive and -sensitive applications, the network has to offer a variety of computing services. More especially, users can offload part of or all computing tasks to the data center [13,14,15]. The data center, nevertheless, is typically built in remote areas, incurring high transmission cost and service latency, and thus failing in meeting the QoS requirements, e.g., high data rate, low latency, and low processing energy consumption [15,16,17,18].

As compared to terrestrial ones, low earth orbit (LEO) satellite networks are typically deployed in the space area with an orbital altitude of 500 to 2000 km. Different from high-orbit and medium-orbit satellites, the LEO satellite’s channel fading and service latency would be greatly reduced. Further, since the LEO satellite network is closer to the ground, it has lower backhaul latency and smaller channel fading, free from the ground terrain [19]. The traditional LEO satellite network is usually limited by the finite onboard capacity. Fortunately, emerging mobile edge computing (MEC) can provide services with low latency, high reliability, high security, and high flexibility by deploying computing and storage resources closer to users [20,21,22]. Assisted by the MEC, LEO satellite edge networks are expected to deploy MEC servers on satellites and cooperate with cloud computing data centers to further reduce energy consumption and task response latency, forming an end-edge-cloud multi-level processing architecture for different business types. That is, MEC servers on satellites can act as edge nodes to provide computing services for the ground terminal, typically with limited capacity. It is also likely that ground terminals offload their tasks to cloud computing data centers [23]. Yet, due to the lack of reliable connectivity to data centers through terrestrial networks, e.g., in remote areas, some tasks have to be forwarded to data centers via the visible LEO satellite. In addition to the cloud-edge-end hierarchical architecture we are investigating, there is a rising trend in integrated continuum architectures. For instance, Trakadas et al. in [24] introduced the meta-operating system reference architecture (RAMOS) to tackle the data surge resulting from IoT proliferation, aiming to establish a dynamic, distributed, and trusted continuum for future data-intensive applications at the edge. Yet, creating a continuum from IoT to the edge and cloud still poses an ongoing challenge [25].

Therefore, we aim to propose a joint computing offloading and resource allocation method in the LEO satellite edge network, based on the parameterized deep Q-network (P-DQN) reinforcement learning (RL), to capture the dynamics in network conditions. The main contributions are listed as follows:

- To better simulate the real LEO network, the dynamic and changeable LEO satellite scenario is defined. The wireless channel with time-varying characteristics is modeled, the communication and computing models under three different offloading strategies are constructed, and the service latency model is obtained.
- The joint computing offloading and resource allocation problem in the LEO satellite edge network is built. Constraints on offloading decisions on processed tasks, on remaining available computing resources, and on power control on both LEO satellites and the cloud server are respectively inferred, followed by the optimization problem formulation.
- For the highly dynamic LEO satellite edge network and the discrete-continuous hybrid action space, an MDP model with parameterized actions is constructed to capture the dynamics in computing offloading, resource allocation, and power control, and the P-DQN RL method is used to maximize the number of accessed tasks.

The rest is organized as follows. In Section 2, a brief summary of existing works on computation offloading and resource allocation in LEO satellite networks is provided. In Section 3, the system model is proposed and the optimization problem is established. In Section 4, the problem is further characterized by the parameterized Markov process and solved by the P-DQN. In Section 5, simulation experiments are conducted to verify the algorithm performance. Finally, Section 6 concludes the work and provides an outlook on future endeavors.

## 2. Related Work

In ground edge networks, there have been many works on joint computation offloading and resource allocation. Yan et al. in [26] introduced the multi-user edge network scenario, considered the task dependency among users, and formulated it as a mixed-integer program, to optimize both task offloading and power control decisions, for the minimization of a weighted sum of energy consumption and latency. Likely, a multi-user multi-task network scenario was presented in [27], by formulating it as a mixed integer program and considering the service caching, computation offloading, and resource allocation, to minimize the weighted sum of latency and energy consumption. Besides, Wu et al. in [28] introduced a multi-cell MEC-assisted network, developed an analytical model to decouple power control and computing resource allocation, and proposed heuristics. Tan et al. in [29] studied the multi-user cooperative MEC network based on orthogonal frequency division multiple access (OFDMA), and formulated the collaborative decision making, computation offloading, and resource allocation as a mixed nonlinear program. In particular, to minimize the total energy consumption of devices, a two-stage alternating framework is proposed to decompose the collaborative problem into two layers, of which the first one is the offloading decision generation method based on an ant colony system, and the second one is the resource allocation method based on deep *Q* network, to obtain the optimal power control, subcarrier assignment, and computing resource allocation, given offloading decisions. Acknowledging the importance of energy efficiency (EE) optimization, Ruan et al. in [30] focused on the energy-efficient power allocation in cognitive satellite–terrestrial networks. Besides, optimal power allocation schemes for both non-real-time and real-time applications were addressed in [31], for optimizing the EE of cognitive satellite users. Spantideas et al. in [32] introduced a power configuration algorithm based on deep Q-Learning for 5G cells, thereby optimizing both EE and throughput adequacy. Likewise, the joint power allocation and user association in wireless heterogeneous networks using the DRL was proposed in both [33,34]. However, due to technical limitations, EE optimization is not covered in the current work, and would be encapsulated in future work.

Moreover, unmanned aerial vehicle (UAV)-assisted MEC begins to emerge, bringing more sophisticated computation offloading issues. Li Bin et al. in [35] utilized the double deep Q-network algorithm to investigate the task offloading problem in UAV-enabled MEC with the digital twin, by optimizing the mobile terminal user association, UAV trajectory planning, transmission power distribution, and computing resource allocation, thereby minimizing the system energy consumption. Likewise, the UAV-assisted MEC was also proposed to support resource-intensive applications in [36]. More precisely, by introducing the digital twin-empowered MEC network with multiple UAVs and one ground base station, the multi-agent proximal policy optimization is used to save energy.

As compared to ground edge networks and UAV-assisted MEC, the research on computation offloading and resource allocation in LEO satellite edge networks is still in the preliminary stage. Considering the high dynamics in the LEO satellite environment, how to offload tasks to nodes with abundant resources, and how to allocate resources to those offloaded tasks have become the challenges. Qiu et al. in [37] proposed a software-defined space-ground integrated network framework for the management and orchestration of caching and computing resources, using deep *Q*-learning methods. Xu et al. in [38] proposed a satellite-assisted maritime network architecture on edge computing, using deep *Q* learning to minimize the total service latency. In aforementioned both works, although cloud servers with substantial computing resources are mentioned, both network models only include the LEO satellite layer and base station layer, and the explicit incorporation of cloud servers into the model is missing. In contrast, we aim to propose the multi-tier cloud-edge-end architecture, encompassing computing-capable end users, MEC-assisted LEO satellites, and the cloud server with rich resources, thereby providing users with a wider range of offloading options and access opportunities.

Furthermore, Cheng et al. in [39] used the deep reinforcement learning (DRL) method to learn the optimal offloading decision dynamically in an air-ground integrated edge network, meanwhile proposing heuristics to solve the mixed integer program of joint computing resource allocation and task scheduling. Cui et al. in [40] respectively used the Lagrange multiplier and DRL methods to optimize the service latency, provided the resource allocation is given. Wang et al. in [41] likely decomposed the joint problem into two sub-problems, using the Lagrange multiplier method for communication and computing resource allocation provided the computation offloading is preserved. The cooperative offloading problem in LEO satellite Internet of Things (IoTs) was studied in [42], where LEO satellites forward tasks to ground MEC servers, and the weighted latency and energy minimization problem is designed as a partially observable MDP (POMDP), followed by the multi-agent DRL framework.

In brief, existing works have extensively explored the challenges of computation offloading and resource allocation in either ground-edge networks [26,27,28,29] or UAV-assisted MEC [35,36]. Yet, unlike previous studies on satellite edge networks [39,40,41,42], we consider the more precise description of dynamic characteristics of the network, including the relative position variation between LEO satellites and ground terminals, together with time-varying channel fading. Further, in existing RL works addressing computation offloading in LEO satellite edge networks, it is common to decompose the original problem into two sub-problems [37,38,39,40,41,42]. In contrast, we resort to the P-DQN method, thereby handling the hybrid action space and offering a more integrated solution, without the intricate problem decomposition.

## 3. System Model and Problem Description

### 3.1. LEO Satellite Edge Network Model

**Figure 1:**
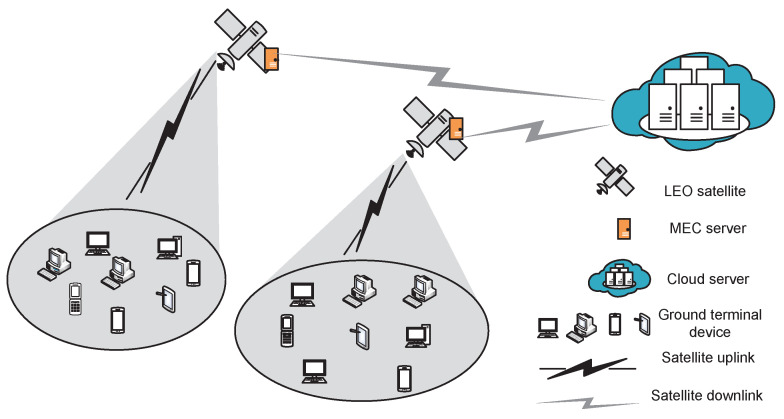
LEO satellite edge networks.

The LEO satellite edge network model is shown below in Figure 1.

As shown in Figure 1, the network includes multiple ground terminals, several LEO satellites equipped with edge servers, and one cloud server. Let L={1,2,…,L} be the satellite set, with *L* as the total number of satellites. Designate that LEO satellites use Ka-band (27–40 GHz) to provide access for ground terminals, and each satellite only takes charge of terminals within its coverage. Moreover, let K={1,2,…,K} be the division of *K* regions, and then let $I_{k}=\left\{1,2,\ldots ,I_{k}\right\}$ be the set of terminals in region *k*. Assume that all terminals within one coverage access the same satellite (each terminal, yet, can only access one satellite at one time), while each satellite can serve multiple terminals simultaneously. Since terminals within the same coverage have similar distances, the channel states (between terminals and LEO satellites) are almost identical, and the time division multiple access (TDMA) can work to avoid multi-user interference within the region. Next, let *M* be the cloud server. Due to the long distance and sophisticated geographical conditions, a direct connection between *M* and terminals cannot be established. Thus, the service flow from terminals must be forwarded through LEO satellites to *M*.

To better depict dynamics in the network due to the random arrival of tasks, the system time can be discretized into successive time slots with equal length, i.e., T={1,2,…,T}. Let $R_{k,i}^{\bar{t}}$ be the new arrival task of terminal *i* in region *k* during time slot $\bar{t}\in T$, and $l_{k}\in L$ be the satellite covering region *k*. Further, all tasks are latency sensitive, and denote $t_{\max}$ as the maximum tolerance latency per task and $c_{k,i}^{\bar{t}}$ as the size per packet to be processed by the task, respectively. Besides, only consider tasks surviving for several time slots, the duration of which is far less than the continuous coverage duration of LEO satellites for one region (which is set as about 9 min in [43]). As such, almost all tasks considered can be terminated (accessed or failed) within the LEO satellite covering the duration, and thus the handover and its associated unstable connections are reasonably ignored. Note that, due to the insufficient computing capability and limited resources in ground terminals, a larger processing latency would be incurred locally. In contrast, when offloading tasks to LEO satellites, a relatively larger propagation latency is preferred.

Thus, there are three options for the terminal to process task $R_{k,i}^{\bar{t}}$, and the latency depends on the computation offloading mode. Let $X_{k,i}^{\bar{t},t}=\left\{x_{k,i}^{\bar{t},t,1},x_{k,i}^{\bar{t},t,2},x_{k,i}^{\bar{t},t,3}\right\}$ be the offloading set of task $R_{k,i}^{\bar{t}}$ at slot *t* (originating at $\bar{t}$ and not yet completely terminating until *t*), where $x_{k,i}^{\bar{t},t,1},x_{k,i}^{\bar{t},t,2},x_{k,i}^{,\bar{t},t,3}\in \left\{0,1\right\}$ and $x_{k,i}^{\bar{t},t,1}+x_{k,i}^{\bar{t},t,2}+x_{k,i}^{\bar{t},t,3}=1$, i.e., each task $R_{k,i}^{\bar{t}}$ can only choose one processing method. In particular, $x_{k,i}^{\bar{t},t,1}=1$, $x_{k,i}^{\bar{t},t,2}=1$ and $x_{k,i}^{\bar{t},t,3}=1$ denote that the task is processed locally, offloaded to the satellite, and offload to the cloud server, respectively.

### 3.2. Channel Model

Due to the high mobility of LEO satellites, the relative position between satellites and ground terminals changes rapidly, and so do the free space loss, atmospheric fading, and many other factors involved in the satellite–terrestrial link [44]. As shown in Figure 1, the link from terminals to the LEO satellite is named as satellite uplink, and that from the LEO satellite to the cloud server is named as the satellite downlink. Note that when returning processing results to terminals, the transmission latency (and thus the link) is ignored due to the small-sized result. Thus, when offloading task $R_{k,i}^{\bar{t}}$ to satellites, the data transmission only goes through the satellite uplink; when offloading it to the cloud server through the LEO satellite, the data transmission must go through the satellite uplink first and then through the satellite downlink.

As stated in Section 3.1, the channel state of all terminals in the same satellite coverage is almost identical, so the satellite uplink channel state $g_{k}^{t,L}$ in region *k* at slot *t* can be defined as

(1) $$g_{k}^{t,L}=G_{s}^{E}\cdot G_{k}^{t,L}\cdot G_{r}^{L},$$

where $G_{s}^{E}$ denotes the transmit antenna gain of terminals, $G_{k}^{t,L}$ is the channel fading between region *k* and the associated satellite at slot *t*, and $G_{r}^{L}$ represents the receiving antenna gain of satellites. In particular, the channel fading between terminals and satellites generally includes free space path loss, atmospheric fading, and small-scale fading (obeying the Rician distribution) [45], i.e.,

(2) $$G_{k}^{t,L}={\left(\frac{c}{4\pi d_{k}^{t,L}f_{e}}\right)}^{2}\cdot \Phi \left(\alpha^{t,L}\right)\cdot \psi ,$$

where *c* represents the light speed, $d_{k}^{t,L}$ is the distance between region *k* and its access satellite at slot *t*, $f_{e}$ denotes the carrier frequency, $\Phi \left(\alpha^{t,L}\right)$ specifies the atmospheric fading, and ψ is the Rician distributed small-scale fading. More precisely, atmospheric fading $\Phi \left(\alpha^{t,L}\right)$ is expressed as

(3) $$\Phi \left(\alpha^{t,L}\right)=10^{\left(\frac{3\delta }{10\sin\alpha^{t,L}}\right)},$$

where $\sin\alpha^{t,L}=H/d_{k}^{t,L}$, *H* is the orbital altitude of LEO satellites, and δ is the attenuation through rain and clouds, separately. The Rayleigh fading channel models (e.g., in [46,47]) is not used throughout.

Further, the channel state of satellite downlink between LEO satellites and the cloud server *M* for region *k* at slot *t* is represented as $g_{k}^{t,M}$ as

(4) $$g_{k}^{t,M}=G_{s}^{L}\cdot G_{k}^{t,M}\cdot G_{r}^{M},$$

where $G_{s}^{L}$ is the transmit antenna gain of LEO satellites, $G_{k}^{t,M}$ is the channel fading between LEO satellites and the cloud server for region *k* at slot *t*, and $G_{r}^{M}$ is the receiving antenna gain of cloud server. Likewise, the channel fading of satellite downlink also includes free space path loss, atmospheric fading and Rician distributed small-scale fading, respectively, i.e.,

(5) $$G_{k}^{t,M}={\left(\frac{c}{4\pi d_{k}^{t,M}f_{l}}\right)}^{2}\cdot \Phi \left(\alpha^{t,M}\right)\cdot \psi ,$$

where $d_{k}^{t,M}$ is the distance between *M* and satellite $l_{k}$, $f_{l}$ is the carrier frequency of LEO satellites, and $\Phi \left(\alpha^{t,M}\right)$ is the atmospheric fading, i.e.,

(6) $$\Phi \left(\alpha^{t,M}\right)=10^{\left(\frac{3\delta }{10\sin\alpha^{t,M}}\right)},$$

with $\sin\alpha^{t,M}=H/d_{k}^{t,M}$.

### 3.3. Latency and Satisfied Task Model

First, when locally processing task $R_{k,i}^{\bar{t}}$ on terminals, the total service latency includes only the processing latency, i.e., $T_{k,i}^{\bar{t},\mathrm{tol}}=c_{k,i}^{\bar{t}}/C_{k,i}^{\bar{t},E}$, where $c_{k,i}^{\bar{t}}$ represents the packet size of $R_{k,i}^{\bar{t}}$, and $C_{k,i}^{\bar{t},E}E$ is the remaining constant computing resource always allocated to $R_{k,i}^{\bar{t}}$, respectively. If $T_{k,i}^{\bar{t},\mathrm{tol}}\leq t_{\max}$, then $R_{k,i}^{\bar{t}}$ is satisfied; otherwise, it fails.

Second, when offloading $R_{k,i}^{\bar{t}}$ to satellite $l_{k}$, the total service latency consists of processing, transmission, and propagation ones. The processing latency is $c_{k,i}^{\bar{t}}/C_{k,i}^{\bar{t},L}$, where $C_{k,i}^{\bar{t},L}$ is the computing resource always allocated to $R_{k,i}^{\bar{t}}$ by $l_{k}$, and the propagation latency is $2d_{k}^{\bar{t},L}/c$. Further, the transmission rate in the satellite uplink becomes

(7) $$s_{k,i}^{\bar{t},t,L}=W_{k}^{L}\log_{2}\left(1+\frac{P_{k,i}^{\bar{t},L}g_{k}^{t,L}}{\sum_{m=1,m\neq k}^{K}\sum_{j=1}^{I}P_{m,j}^{\bar{t},L}g_{m}^{t,L}+\sigma^{2}}\right),$$

where $W_{k}^{L}$ represents the link bandwidth allocated to region *k*, $s_{k,i}^{\bar{t},t,L}$ is the transmission rate of $R_{k,i}^{\bar{t}}$ (originating at slot $\bar{t}$ and not yet transmitted completely until slot *t*) at *t*, $P_{k,i}^{\bar{t},L}$ is the transmit power allocated to $R_{k,i}^{\bar{t}}$ and we assume that $P_{k,i}^{\bar{t},L}$ does not vary across slots for simplicity, $\sum_{m=1,m\neq k}^{K}\sum_{j=1}^{I}P_{m,j}^{\bar{t},L}g_{m}^{t,L}$ is the interference power caused by other regions except *k* at slot *t*, and $\sigma^{2}$ is the noise power, respectively. Since the channel state varies across slots, the transmission rate also changes. Besides, the remaining data (not yet transmitted) of $R_{k,i}^{\bar{t}}$ becomes

(8) $$z_{k,i}^{\bar{t},t}=\left\{\begin{array}{c} c_{k,i}^{\bar{t}}-s_{k,i}^{\bar{t},t,L}\cdot \tau ,\;t=\bar{t}\\ z_{k,i}^{\bar{t},t-1}-s_{k,i}^{\bar{t},t,L}\cdot \tau ,\;t>\bar{t} \end{array}\right.,$$

where τ is the size per slot. At the beginning of each slot *t*, judgments are made depending on the state of $R_{k,i}^{\bar{t}}$. Define variables $\eta_{k,i}^{\bar{t},t}$ to represent the condition of $R_{k,i}^{\bar{t}}$ (originating at slot $\bar{t}$) at current slot *t*, i.e.,

(9) $$\eta_{k,i}^{\bar{t},t}=\left\{\begin{array}{cc} 1, & \mathrm{if}\;T_{k,i}^{\bar{t},\mathrm{tol}}\leq t_{\max}\\ 0, & \mathrm{if}\;T_{k,i}^{\bar{t},\mathrm{tol}}>t_{\max}\;\mathrm{or}\;T_{k,i}^{\bar{t},\mathrm{genr}}\geq t_{\max}\\ -1, & \mathrm{if}\;T_{k,i}^{\bar{t},\mathrm{genr}}<t_{\max} \end{array}\right.,$$

where $T_{k,i}^{\bar{t},\mathrm{tra}}$ is the traversed latency from $\bar{t}$ to *t* of unfinished task $R_{k,i}^{\bar{t}}$. $\eta_{k,i}^{\bar{t},t}=1$, $\eta_{k,i}^{\bar{t},t}=0$ and $\eta_{k,i}^{\bar{t},t}=-1$ indicates that $R_{k,i}^{\bar{t}}$ is exactly finished and judged to be satisfied, that $R_{k,i}^{\bar{t}}$ is judged to fail, and that the judgment has to be postponed to next slot, all at slot *t*. The explicit judgment on satisfied conditions of tasks are shown in Algorithm 1.

Third, when offloading $R_{k,i}^{\bar{t}}$ to the cloud server *M*, the total service latency consists of processing, transmission, and propagation ones as well. The processing latency is $c_{k,i}^{\bar{t}}/C_{k,i}^{\bar{t},M}$, where $C_{k,i}^{\bar{t},M}$ is the computing resource always allocated to $R_{k,i}^{\bar{t}}$, and the propagation latency is $(2d_{k}^{\bar{t},L}+2d_{k}^{\bar{t},M})/c$. Further, the transmission rate in the satellite downlink becomes

(10) $$s_{k,i}^{\bar{t},M}=W_{k}^{M}\log_{2}\left(1+\frac{P_{k,i}^{\bar{t},M}g_{k}^{t,M}}{\sum_{m=1,m\neq k}^{K}\sum_{j=1}^{I}P_{m,j}^{\bar{t},M}g_{m}^{t,M}+\sigma^{2}}\right),$$

where $W_{k}^{M}$ represents the link bandwidth allocated to region *k*, and $\sum_{m=1,m\neq k}^{K}\sum_{j=1}^{I}P_{m,j}^{\bar{t},M}g_{m}^{t,M}+\sigma^{2}$ is the interference power caused by other regions except *k* at slot *t*. Assume that the satellite works in the full-duplex mode when forwarding the data from terminals to *M* via satellites, the transmission rate of $R_{k,i}^{\bar{t}}$ becomes $s_{k,i}^{\bar{t},t,M^{{}^{\prime }}}=\min\left\{s_{k,i}^{\bar{t},t,L},s_{k,i}^{\bar{t},t,M}\right\}$. Since the channel state varies across slots, the transmission rate also changes, and the judgment on satisfied conditions resembles Algorithm 1, with a slight difference on the data rate calculation.
**Algorithm 1** Judgment on satisfied conditions of task $R_{k,i}^{\bar{t}}\left(\bar{t}\leq t\right)$ at slot *t***Input:**  unfinished task $R_{k,i}^{\bar{t}}\left(\bar{t}\leq t\right)$ **Output:** judgment result $\eta_{k,i}^{\bar{t},t}$  1:Initialize $\eta_{k,i}^{\bar{t},t}=-1$  2:**while** $\eta_{k,i}^{\bar{t},t}=-1$ **do**  3:    Calculate the size of remaining data $z_{k,i}^{\bar{t},t}$  4:    **if** $z_{k,i}^{\bar{t},t}\leq 0$ **then**  5:        Obtain the total service latency: $T_{k,i}^{\bar{t},\mathrm{tol}}=\left(c_{k,i}^{\bar{t}}/C_{k,i}^{\bar{t},L}\right)+\left(t-\bar{t}+1\right)\cdot \tau +\left(2d_{k}^{\bar{t},L}/c\right)$  6:        **if** $T_{k,i}^{\bar{t},\mathrm{tol}}\leq t_{\max}$ **then**  7:           $\eta_{k,i}^{\bar{t},t}=1$  8:        **else**  9:           $\eta_{k,i}^{\bar{t},t}=0$10:        **end if**11:    **else**12:        Calculate the traversed latency: $T_{k,i}^{\bar{t},\mathrm{tra}}=\left(t-\bar{t}+1\right)\cdot \tau +\left(d_{k}^{\bar{t},L}/c\right)$13:        **if** $T_{k,i}^{\bar{t},\mathrm{tra}}\geq t_{\max}$ **then**14:           $\eta_{k,i}^{\bar{t},t}=0$15:        **else**16:           $\eta_{k,i}^{\bar{t},t}=-1$17:        **end if**18:    **end if**19: **end while**


### 3.4. Problem Formulation

Since multiple tasks may arrive at one region at the same slot, they (offloaded to the same satellite) would compete for computing resources. It is prerequisite to jointly optimize computing offloading and resource allocation per slot, to maximize the average number of satisfied tasks over the long run, i.e.,

(11) $$\begin{array}{cc} \; & \max\frac{1}{T}\sum_{t=1}^{T}\sum_{k=1}^{K}\sum_{i=1}^{I}\sum_{\bar{t}=1}^{t}\eta_{k,i}^{\bar{t},t}\\ \; & \begin{array}{cc} s.t. & C1:\;\sum_{i=1}^{I}C_{k,i}^{\bar{t},L}\leq \omega_{k}^{t,L},\forall k,\forall \bar{t},t\\ \; & C2:\;0\leq P_{k,i}^{\bar{t},L}\leq x_{k,i}^{\bar{t},t,2}\cdot P_{k,i,\max}^{L},\;\forall k,\forall i,\forall \bar{t},t\\ \; & C3:\;\sum_{k=1}^{K}\sum_{i=1}^{I}C_{k,i}^{\bar{t},M}\leq \omega^{t,M},\;\forall \bar{t},t\\ \; & C4:\;0\leq P_{k,i}^{\bar{t},M}\leq x_{k,i}^{\bar{t},t,3}\cdot P_{\max}^{M},\;\forall k,\forall i,\forall \bar{t},t\\ \; & C5:\;0\leq C_{k,i}^{\bar{t},L}\leq x_{k,i}^{\bar{t},t,2}\cdot \omega_{k}^{t,L},\;\forall k,\forall i,\forall \bar{t},t\\ \; & C6:\;0\leq C_{k,i}^{\bar{t},M}\leq x_{k,i}^{\bar{t},t,3}\cdot \omega^{t,M},\;\forall k,\forall i,\forall \bar{t},t \end{array} \end{array}$$

where $\omega_{k}^{t,L}$ and $\omega_{}^{t,M}$ are, respectively, the remaining amount of computing resources on $l_{k}$ and *M*, both at slot *t*, and $P_{k,i,\max}^{L}$ and $P_{\max}^{M}$ are the constant maximum transmitting power budget per terminal and per satellite. $C_{1}$ denotes that the sum computing resources allocated to offloaded task does not exceed the remaining ones of $l_{k}$, $C_{2}$ denotes that the transmit power on $R_{k,i}^{\bar{t}}$ does not exceed the budget, $C_{3}$ specifies that the sum computing resources allocated to tasks (offloaded to *M*) are below the remaining capacity, and $C_{4}$ is analogous to $C_{2}$, except that the power is from $l_{k}$ to *M*. Further, $C_{5}$ ($C_{6}$) indicates that only when $x_{k,i}^{\bar{t},t,2}=1$ ($x_{k,i}^{\bar{t},t,3}=1$) holds, $C_{k,i}^{\bar{t},L}$ ($C_{k,i}^{\bar{t},M}$) can take positive values; otherwise, $C_{k,i}^{\bar{t},L}=0$ ($C_{k,i}^{\bar{t},M}=0$) must hold.

## 4. P-DQN-Based Approach

Traditional RL methods such as DQN, actor-critic, and asynchronous actor-critic (A3C) are designed to handle discrete action spaces. The DDPG, on the other hand, is tailored for dealing with continuous actions. To adapt above RL methods to the discrete-continuous hybrid action space, there are two approaches, i.e., either discretizing the hybrid action space, or relaxing it into a continuous one, which would result in a high-dimensional action space. In this work, we use one prevailing architecture, namely P-DQN, which is directly appropriate for hybrid action space without any approximation or relaxation. In particular, existing P-DQN frameworks (which are predominantly used in the game control [48]), are enabled to address the computation offloading in LEO satellite edge networks. To adapt the classical P-DQN, we integrate offloading decisions with resource allocation into one hybrid action space. More precisely, the MDP with parameterized action space has to be constructed, followed by assessing the satisfaction of tasks and establishing the deferred reward function. This type of parameterized action space facilitates the maximization of satisfied task numbers.

### 4.1. MDP with Parameterized Action Space

The parameterized action MDP (PAMDP) model is an extension of standard MDP [49]. Note that the MDP is represented by a quadruple (S,A,P,R), which are the state space, the action space, the state transition probability set, and the reward function set, separately. In contrast, the PAMDP redefines the discrete-continuous hybrid action space in the MDP, as follows:

(12) $$A=\{\left(v,n_{v}\right)|n_{v}\in N_{v}\;\mathrm{for}\;\mathrm{all}\;v\in V\},$$

where V={1,…,V} is the set of discrete actions, and $N_{v}$ is the set of continuous parameters of each v∈V. A high-level action *v* is first preserved from V, and then the low-level parameter $n_{v}\in N_{v}$ associated with *v* is selected. In particular, the PAMDP model paper can be established as follows:

- **State space:** For each $s^{t}\in S$, define $s^{t}=\left\{Y^{t},Z^{t},{\left\{\omega_{k}^{t,L}\right\}}_{k\in K},\omega_{}^{t,M}\right\}$, where $Y^{t}$ and $Z^{t}$, respectively, represent the sets of new arrival tasks and being already processed ones.
- **Parameterized action space:** Define the parameterized action as $A=\left\{a_{k,i}^{\bar{t},t}\right\}$, where $a_{k,i}^{\bar{t},t}=\left\{x_{k,i}^{\bar{t},t,1},x_{k,i}^{\bar{t},t,2}\left(C_{k,i}^{\bar{t},L},P_{k,i}^{\bar{t},L}\right),x_{k,i}^{\bar{t},t,3}\left(C_{k,i}^{\bar{t},M},P_{k,i}^{\bar{t},M}\right)\right\},\bar{t}\leq t$. In particular, $x_{k,i}^{\bar{t},t,1}$, $x_{k,i}^{\bar{t},t,2}$, and $x_{k,i}^{\bar{t},t,3}$ are three types of offloading decisions. For $x_{k,i}^{\bar{t},t,1}$, and the task is processed locally without parameters; for $x_{k,i}^{\bar{t},t,2}$, the task is offloaded to the LEO satellite, the parameters are $C_{k,i}^{\bar{t},L}$ and $P_{k,i}^{\bar{t},L}$; and for $x_{k,i}^{\bar{t},t,3}$, the task is offloaded to the cloud server, and the parameters become $C_{k,i}^{\bar{t},M}$ and $P_{k,i}^{\bar{t},M}$.
- **Transition probability:** A model-free RL architecture is used since both state and action spaces are high-dimensional and we cannot give the precise state transfer.
- **Reward function:** To judge all tasks in $Z^{t}$ per slot, the temporal reward function per task can be defined as $r_{k,i}^{\bar{t},t}$. In particular, when the task is completed in the current slot, $r_{k,i}^{\bar{t},t}$ takes the large positive value; when the task is judged to be transmitted continuously, $r_{k,i}^{\bar{t},t}$ is temporarily set to be zero; and when the task fails, $r_{k,i}^{\bar{t},t}$ is finally set to be negative.

### 4.2. P-DQN Training

The original Bellman equation in Q-learning and DQN is expressed as follows:

(13) $$Q\left(s,a\right)=\underset{r_{t},s_{t+1}}{E}\left[r_{t}+\gamma \max_{a^{\prime }\in A}Q\left(s_{t+1},a^{\prime }\right)|s_{t}=s,a_{t}=a\right].$$

In the hybrid action space A, for each a∈A, the action value function is defined as $Q\left(s,a\right)=Q\left(s,v,n_{v}\right)$. If $v_{t}$ is the discrete action selected at slot *t* and $n_{v_{t}}$ is its associated continuous parameter, then the Bellman equation can be written as

(14) $$Q\left(s_{t},v_{t},n_{v_{t}}\right)=\underset{r_{t},s_{t+1}}{E}\left[r_{t}+\gamma \max_{v\in V}\underset{n_{v}\in N_{v}}{\sup}Q\left(s_{t+1},v,n_{v}\right)|s_{t}=s,a_{t}=\left(v_{t},n_{v_{t}}\right)\right].$$

First, solve $n_{v}^{*}=\arg\sup_{n_{v}^{}\in N_{v}}Q\left(s_{t+1},v,n_{v}\right)$ for all v∈V and then take the maximum $Q(s_{t+1},v,n_{v}^{*})$. When *Q* is fixed, for any s and *v*, $\arg\sup_{n_{v}^{}\in N_{v}}Q\left(s,v,n_{v}\right)$ can be regarded as the mapping $n_{v}^{Q}:S\to N_{v}$, and then (14) becomes

(15) $$Q\left(s_{t},v_{t},n_{v_{t}}\right)=\underset{r_{t},s_{t+1}}{E}\left[r_{t}+\gamma \max_{v\in V}Q\left(s_{t+1},v,n_{v}^{Q}\left(s_{t+1}\right)\right)\;|\;s_{t}=s\right].$$

Similar to DQN, the neural network $Q(s,v,n_{v};w)$ is used to approximate $Q(s,v,n_{v})$ [50], with w as the network weight. For such $Q(s,v,n_{v};w)$, the deterministic policy network $n_{v}\left(\cdot ;\theta \right):S\to N_{v}$ can approximate $n_{v}^{Q}\left(s\right)$, with θ as the policy network weight. That is, given w, for each v∈V, θ is obtained as

(16) $$Q\left(s,v,n_{v}\left(s;\theta \right);w\right)\approx \underset{n_{v}\in N_{v}}{\sup}Q\left(s,v,n_{v};w\right),$$

where w is estimated by minimizing the mean square Bellman error by the gradient descent. In the *t*-th step (slot) and with the multi-step algorithm with *j*, the *j*-th step’s target becomes

(17) $$y_{t}=\sum_{i=0}^{j-1}\gamma^{i}r_{t+i}+\gamma^{j}\max_{v\in V}Q(s_{t+j},v,n_{v}\left(s_{t+j},\theta_{t}\right);w_{t}).$$

Then, the least squared loss function is used to train w as follows:

(18) $$L_{t}\left(w\right)=\frac{1}{2}{\left[Q\left(s_{t},v_{t},n_{v_{t}};w\right)-y_{t}\right]}^{2}.$$

In particular, the objective is to find θ maximizing $Q(s,v,n_{v}\left(s;\theta \right);w)$ when w is fixed, whereas conventional loss functions typically require minimization. Therefore, a negative sign is added in (19) to formulate it as a loss function, allowing us to simultaneously maximize the objective while minimize the loss function, as follows:

(19) $$L_{t}\left(\theta \right)=-\sum_{v=1}^{V}Q(s_{t},v,n_{v}\left(s_{t};\theta \right);w_{t})/V.$$

Next, the data in the experience replay pool is used to obtain the stochastic gradient $\nabla_{w}L_{t}\left(w_{t}\right)$ and $\nabla_{\theta }L_{t}\left(\theta_{t}\right)$, and both weights are updated as follows:

(20) $$w_{t+1}=w_{t}-\beta_{t}\nabla_{w}L_{t}\left(w_{t}\right),$$

and

(21) $$\theta_{t+1}=\theta_{t}-\zeta_{t}\nabla_{\theta }L_{t}\left(\theta_{t}\right),$$

where $\beta_{t}$ and $\zeta_{t}$, respectively, denote the step sizes for updating parameters w and θ.

**Figure 2:**
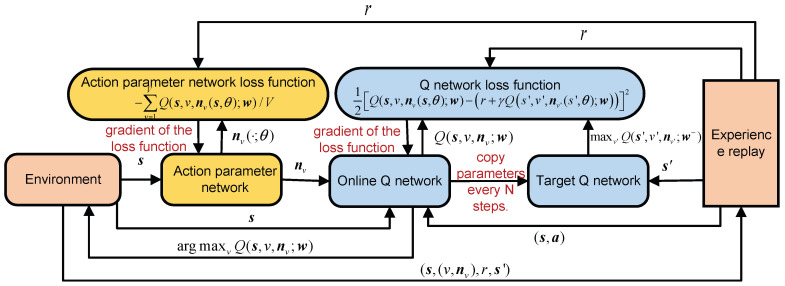
Flowchart for joint computation offloading and resource allocation with P-DQN.

Till now, the joint computation offloading and resource allocation algorithm for LEO edge networks with P-DQN is listed in Algorithm 2, together with the flowchart in Figure 2. Apparently, the P-DQN is an online and off-policy RL method.
**Algorithm 2** Joint computation offloading and resource allocation with P-DQN**Input:** step sizes ${\left\{\beta_{t}\right\}}_{t=1}^{T}$ and ${\left\{\zeta_{t}\right\}}_{t=1}^{T}$, exploration rate ε and batch size *U*.
  1:Initialize the simulation environment parameter settings.  2:Initialize the network parameters ω and θ, and empty the replay buffer *D*.  3:**for** t=1,2,…,T **do**  4:    Calculate action parameters (computing resources and power): $n_{v}\left(s_{t},\theta_{t}\right)\to n_{v}$.  5:    Select the action $a_{t}=\left(v_{t},n_{v_{t}}\right)$ according to the ϵ-greedy strategy:
$$a_{t}=\left\{\begin{array}{cc} \left(v_{t},n_{v_{t}}\right)\;\mathrm{such}\;\mathrm{that}\;v_{t}=\arg\max_{v\in V}Q\left(s_{t},v,n_{v};w_{t}\right), & \;\mathrm{with}\;\mathrm{probability}\;1-\varepsilon \\ \mathrm{sample}\;\mathrm{an}\;\mathrm{action}\;\mathrm{in}\;A\;\mathrm{randomly}. & \mathrm{with}\;\mathrm{probability}\;\varepsilon \end{array}\right.$$  6:    Execute $a_{t}$ and store the tuple $\left(s_{t},a_{t},r_{t},s_{t+1}\right)$ into *D*.  7:    Randomly sample *U* tuples $\left(s_{u},a_{u},r_{u},s_{u+1}\right)$ from *D*, u=1,2,…,U.  8:    Compute the target *Q* value:
$$\begin{array}{c} y_{u}=\left\{\begin{array}{cc} r_{u}, & \mathrm{if}\mathrm{is}\_\mathrm{end}\mathrm{is}\mathrm{ture}\\ r_{u}+\max_{v\in V}\gamma Q\left(s_{u+1},v,n_{v}\left(s_{u+1},\theta_{t}\right);w_{t}\right). & \mathrm{otherwise} \end{array}\right. \end{array}$$  9:    Use the experience replay to obtain the stochastic gradients $\nabla_{w}L_{t}\left(w_{t}\right)$ and $\nabla_{\theta }L_{t}\left(\theta_{t}\right)$.10:    Update the parameters w and θ based on (20) and (21).11: **end for**


## 5. Simulations and Results Analysis

### 5.1. Parameter Settings

Experiments are conducted on a simulation server equipped with one NVIDIA GeForce RTX 3060 graphics card, one 12th generation 6-core processor, and two 8 GB RAM modules. The software environment involves Python 3.9.13, PyTorch 1.13.0, and satellite tool kit (STK 11.6). Detailed parameter settings are presented in Table 1.

In particular, to acquire position information of LEO satellites across 100 time slots, three LEO satellites are simulated at an orbital height of 900 Km using STK, thereby obtaining position information reports. The CPU’s computing power is configured at 1000 cycles/bit [51], making the computing resources on the LEO satellite and cloud server reach $3\times 10^{7}$ bit/s and $1\times 10^{8}$ bit/s, respectively [40]. Therefore, the magnitudes of computing resources in the figures below may appear smaller than those in Table 1, but in reality, equivalent, with the only difference in units.

Furthermore, for the *Q* network, the input layer’s dimensionality equals the sum of state space and parameter action dimensions, while the output layer’s dimensionality matches that of the action space. Hidden layers comprise three fully connected ones with 256, 128, and 64 neurons, respectively. Moreover, rectified linear unit (ReLU) activation functions are utilized for each hidden layer for nonlinear mappings. Next, for the parameterized policy network, its input layer matches with the dimensionality of state space, hidden layers mirror those of the *Q* network, and the output layer’s dimensionality corresponds to that of the parameter action, utilizing the hyperbolic tangent (tanh) function as its activation.

### 5.2. Performance Analysis

To validate the effectiveness of the proposed P-DQN method, the following four baseline methods are listed as follows:

(1) Random offloading (RO): Randomly offloading tasks locally, to LEO satellites and to the cloud server [52].
(2) Average resource allocation (ARA): Computing resources on both LEO satellites and the cloud server are evenly shared among offloaded tasks [40].
(3) DQN offloading (DQNO): The DQN is only used for the task offloading [52].
(4) Deep deterministic policy gradient (DDPG) resource allocation (DDPGRA): The DDPG is used to allocate both computing and power resources for already offloaded tasks.

**Figure 3:**
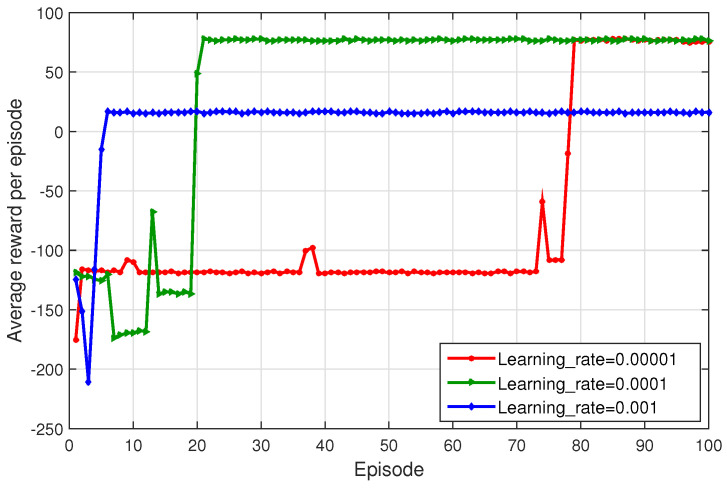
Average reward under different learning rates.

Figure 3 compares the average reward per episode of the proposed method at different learning rates, set to be 0.001, 0.0001, and 0.00001, respectively. Figure 3 clearly shows that the learning rate variation significantly impacts the converged average return and convergence performance. With the learning rate of 0.001, the proposed method begins to converge at episode 10 or so, but next falls into the local optimum with the lower return value. With 0.00001, it does not converge until episode 80 or so. Further, with 0.0001, the proposed method not only demonstrates the improved average return per episode, but also shows a relatively fast convergence rate. It is inferred that a high learning rate can lead to quick convergence but increases the risk of being trapped in the local optimum. Conversely, the lower learning rate results in a smaller step size, thus slowing down the convergence rate to the optimum.

**Figure 4:**
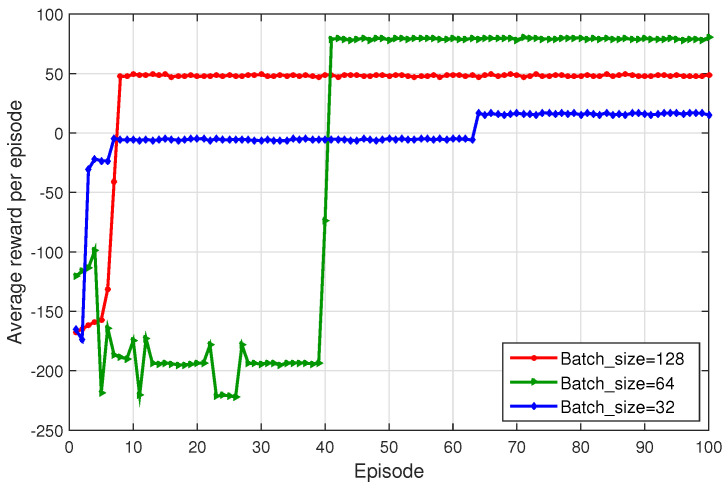
Average return under different batchsize settings.

Figure 4 compares the average return per episode of the proposed method for different batch sizes (32, 64, and 128). The batch size of 32 results in slower convergence and lower average return. Conversely, the batch size of 128 results in a faster convergence rate, but is easily trapped into the local optimum. For 64, both convergence and return values are acceptable.

There, in the sequel, we pick the learning rate of 0.0001 and a sample batch size of 64 for method comparison.

**Figure 5:**
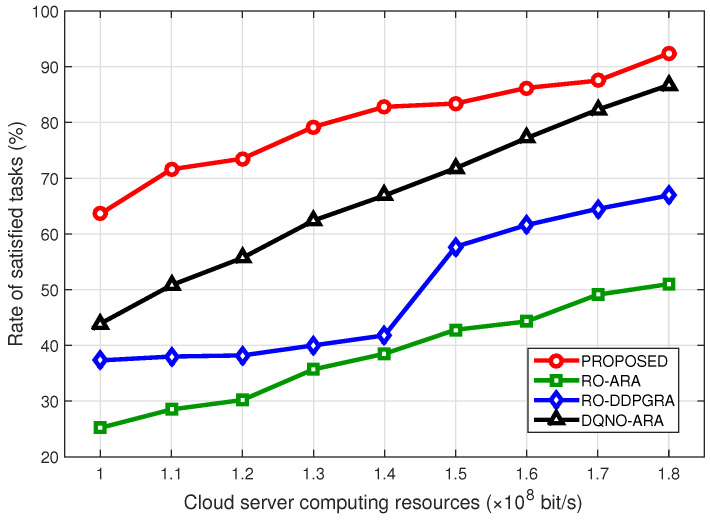
Rate of satisfied tasks under different computing resources budgets in the cloud server.

Figure 5 compares the rate of satisfied tasks under different computing resources budgets in the cloud server. Both RO-ARA and RO-DDPGRA methods use random offloading, thus resulting in a lower access rate when available computing resources are less. Although the rate is improved as computing resources become more, the improvement is limited. This is because that the RO method would not offload more tasks to the cloud server, even with substantial resources. In contrast, both the proposed method and DQNO-ARA tend to prioritize the task of offloading to servers with ample resources. Yet, since the proposed method uses the parameterized continuous resource optimization and DQNO-ARA is just the equal one, the former one obtains the higher rate. Further, as computing resources are gradually increased, the values of both methods continue to grow, yet with the narrowed gap. It is intuitive that more available computing resources would weaken the role of dynamic resource allocation.

**Figure 6:**
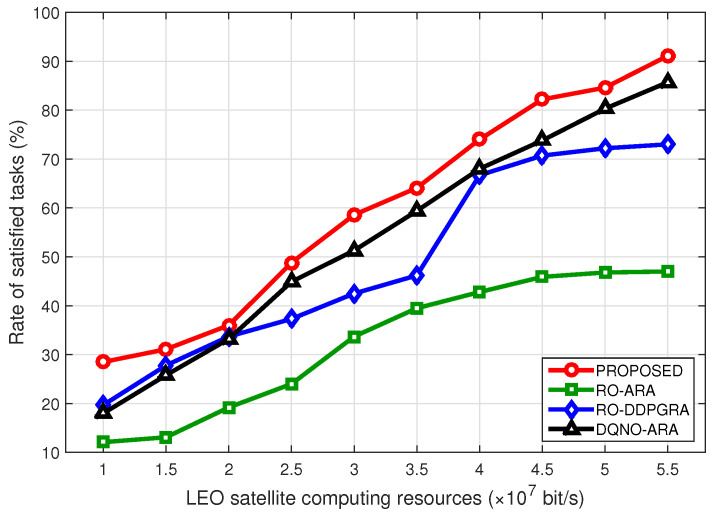
Rate of satisfied tasks under different computing resource budgets of LEO satellites.

Figure 6 illustrates the rate of satisfied tasks at different computing resource budgets in LEO satellites. When available resources are less, all methods exhibit relatively lower satisfied task rates. Both RO-ARA and RO-DDPGRA use random offloading, showing an approximately linear growth with increased resources. By comparison, proposed method and DQNO-ARA consistently achieve higher satisfied rates, surpassing the other two RO methods. Likely, due to the exploitation in dynamic resource allocation, proposed method outperforms DQNO-ARA, nevertheless with the narrowed gap with more resources.

**Figure 7:**
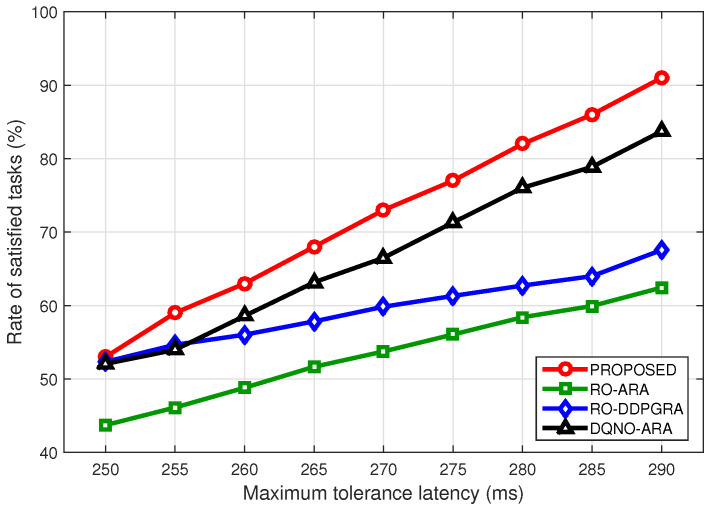
Rate of satisfied tasks under different maximum tolerance latency settings.

In Figure 7, the rate of satisfied tasks under different maximum tolerance latency are compared. As the latency tolerance increases, the rates of all methods show an approximately linear growth. Given smaller tolerance, the rates of three other methods are almost identical except for the RO-ARA method. However, as the latency tolerance increases, proposed method exhibits significant advantages. In particular, at the tolerance of 290 ms, proposed method is improved by 7%, 22%, and 27% over three other ones, respectively. Note that even at the tolerance of 290 ms, the rates of satisfied tasks of RO-ARA and RO-DDPGRA are still relatively lower, due to the fact that some tasks are randomly offloaded locally. As such, there tasks cannot be completed within the tolerance, owing to the limited computing resources in ground terminals.

**Figure 8:**
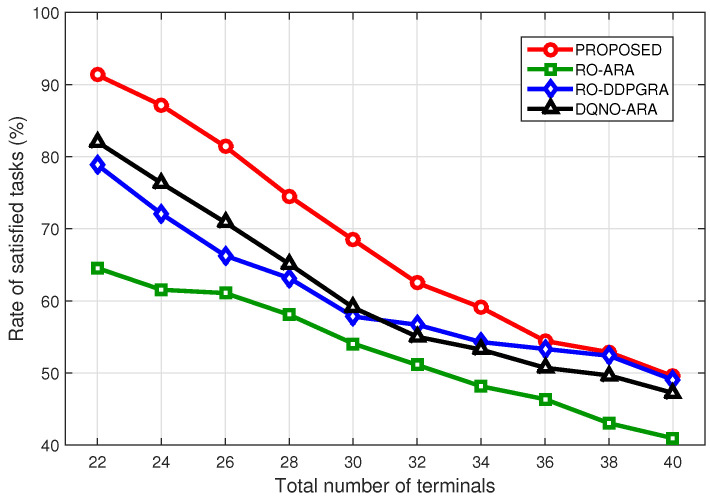
Rate of satisfied tasks under different terminal numbers.

Figure 8 further compares the rate of satisfied tasks under different numbers of terminals. As the number increases, the rates for all methods show a declining trend. In particular, given more terminals, the RO-DDPGRA method surpasses the DQNO-ARA, suggesting that more existing terminals would make the resource allocation dominate computation offloading. More precisely, when the terminal number reaches 40, except for the RO-ARA, the rates of three other methods become close. That is, given the excessive terminal number, both computation offloading and resource allocation begin to take no effects on the performance. Nevertheless, given either more or smaller existing terminals, proposed method always outperforms benchmark ones.

**Figure 9:**
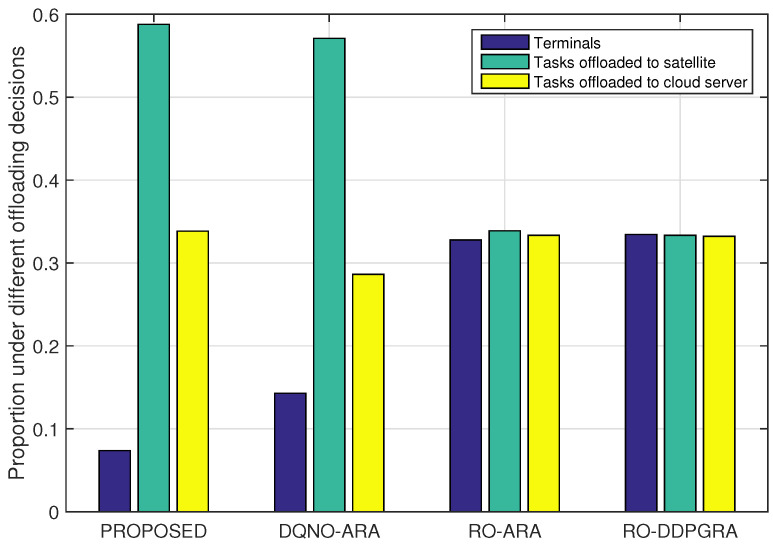
Proportion of offloaded tasks under different approaches.

**Figure 10:**
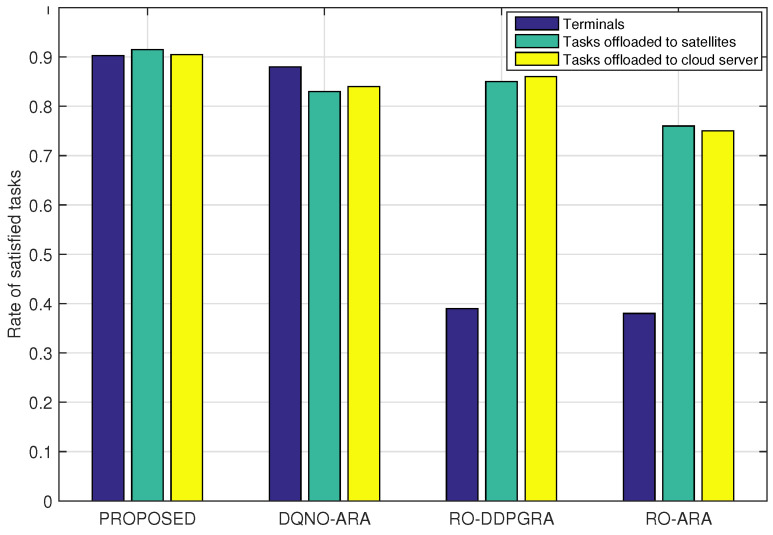
Rate of satisfied tasks in terminals, satellites, and cloud server.

Figure 9 and Figure 10 illustrate the proportion of offloaded tasks under different approaches, and the rate of satisfied tasks in terminals, satellites and the cloud server, respectively. Both figures reveal that in RO-ARA and RO-DDPGRA methods, both employing random offloading, the number of offloaded tasks is equal to that of locally executed ones. In Figure 10, the rate of satisfied tasks in terminals is consistently below 40%. However, the RO-DDPGRA method shows around 10% higher rate for tasks offloaded to satellites, and 8% higher for tasks offloaded to the cloud server, both over the RO-ARA, owing to RO-DDPGRA. In particular, RO-DDPGRA adopts the DDPG, which can deterministically optimize resources to tasks offloaded to the same satellite (or cloud server), while RO-ARA only evenly distributes resources. In contrast, both PROPOSED and DQNO-ARA methods have significantly lower rates of tasks locally executed, and noticeably higher rates of tasks offloaded. Moreover, PROPOSED exhibits approximately 8% (and 6%) higher satisfaction for tasks offloaded to satellites (and the cloud server), respectively, than DQNO-ARA, due to the difference in the resource allocation. Likewise, PROPOSED can allocate resources in line with the parameterized continuous action, while DQNO-ARA can only distribute resources equally. Note that RO-DDPGRA achieves higher satisfaction for tasks offloaded than the DQNO-ARA; yet, due to the randomness in the offloading decisions, the overall rate of satisfied tasks of the RO-DDPGA is constrained and falls below that of the DQNO-ARA method.

## 6. Conclusions and Future Work

This work has investigated the application of P-DQN RL method to the joint computation offloading and resource allocation problem in LEO satellite edge networks. Unlike the discrete action space-based method (e.g., DQN) and the continuous action space-based one (e.g., DDPG), the P-DQN method takes effects in the mixed discrete-continuous space, without approximating the mixed space into the discrete one or relaxing it into the continuous one. Thus, this work considered the time-varying channel characteristics, and formulated the power control, computation offloading, and resource allocation to maximize the rate of satisfied tasks over the long run. To solve it, the PAMDP model was used to capture the dynamics in LEO satellite edge networks, using the parameterized continuous action. Finally, the effectiveness of proposed method was verified through simulations, showing that it not only has a faster convergence rate, but also outperforms existing methods in terms of the rate of satisfied tasks. In future work, we will consider issues such as unstable connections between terminals and satellites (i.e., satellite handovers), and long-run optimization of EE.

## Figures and Tables

**Table 1:** Simulation parameters.

Parameters	Value
Length per time slot	0.1 s
Slot number per episode	100
Task size	$\left[8\times 10^{4},1.2\times 10^{5}\right]$ bits
Number of LEO satellites	3
LEO satellite orbit altitude	900 Km
Maximum transmit power per LEO satellite	100 w
Maximum transmission power per ground terminal	20 w
Carrier frequency of Ka-Band	30 GHz
Link bandwidth	25 MHz
Number of terminals in region 1	14
Number of terminals in region 2	12
Number of terminals in region 3	8
Noise power spectral density	−174 dBm/Hz
Computing resources per LEO satellite	$3\times 10^{10}$ cycle/s
Computing resources of cloud server	$1\times 10^{11}$ cycle/s
Maximum tolerance latency	260 ms
Discounting factor	0.9

## Data Availability

The data presented in this study are available on request from the corresponding author.
